# Crystal structure of 4,4-di­bromo-1-(3,4-di­meth­oxy­phen­yl)-2-aza­buta-1,3-diene-1-carbo­nitrile

**DOI:** 10.1107/S2056989016011075

**Published:** 2016-07-22

**Authors:** Marwa Chaabene, Abderrahim Khatyr, Michael Knorr, Moheddine Askri, Yoann Rousselin, Marek M. Kubicki

**Affiliations:** a Laboratoire de Chimie Hétérocyclique, Produits Naturels et Réactivité (LR11ES39), Département de Chimie, Faculté des Sciences de Monastir, Tunisia; b Institut UTINAM UMR CNRS 6213, University of Franche-Comté, 16 route de Gray, 25030 Besançon, France; c ICMUB UMR CNRS 6302, University of Bourgogne, 9 avenue Alain Savary, 21078 Dijon, France

**Keywords:** crystal structure, substituted 2-aza­buta-1,3-diene, weak hydrogen bonding, halogen bonding, π–π inter­actions

## Abstract

The title compound, C_12_H_10_Br_2_N_2_O_2_, represents an example of a planar π-con­jugated 2-aza­butadiene mol­ecule, which is both an inter­esting starting material for further organic transformations and a potential ligand in organometallic coordination chemistry. Its metric mol­ecular parameters are typical for the family of 2-aza­buta-1,3-dienes not substituted at the (CH) 3-position. In the crystal, the almost planar (r.m.s. deviation = 0.0658 Å) aza­diene mol­ecules form one-dimensional double-wide ribbons through inter­molecular halogen bonds (C—Br⋯O and C—Br⋯Br—C), which then stack in a slipped manner through weak C—H⋯Br and π–π inter­actions to generate a three-dimensional network.

## Abstract 2

The substitution of the aryl group at *trans* position with respect to the N atom in the imine part of 4,4-dihalogeno-1,1-aryl-2-aza­buta-1,3-dienes [Ar_2_C=N—C(H)=C*X*_2_] by a CN group allows to get an almost perfectly planar mol­ecule.

## Chemical context   

**Figure 1:**
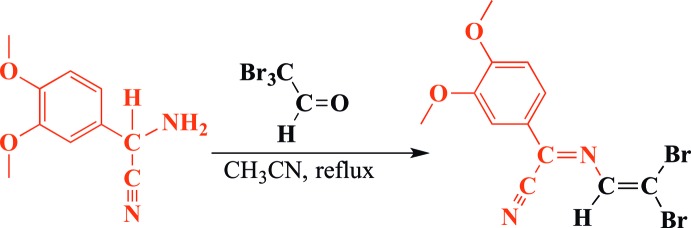
The reaction scheme for the synthesis of (1).

In the context of our inter­est in developing novel π-conjugated di­thio­ether compounds as ligands for coordination chemistry and further organic transformations, we have re­ported on the synthesis and crystal structure of 4,4-di­chloro-1,1-diphenyl-2-aza­buta-1,3-diene [Ph_2_C=N—C(H)=CCl_2_] and its conversion to [Ph_2_C=N—C(H)=C(SR)_2_] and [Ph_2_C=N—C(H)=C(OPh)_2_] by reaction with thiol­ates NaS*R* or NaOPh, respectively (Jacquot *et al.*, 1999 ▸, 2000 ▸; Jacquot-Rousseau *et al.*, 2006 ▸; Kinghat *et al.*, 2016 ▸). Several crystal structures of these mol­ecules/ligands and their derived transition metal complexes reveal that despite the overall planarity of the π-conjugated chain, one aryl group of the –N=CPh_2_ imine segment is tilted with respect to the aza­butadienic array (Jacquot *et al.*, 1999 ▸; Knorr *et al.*, 2003 ▸; Kinghat *et al.*, 2008 ▸). To circumvent this feature and to modulate the stereoelectronic properties, we examined other synthetic strategies for the synthesis of 2-aza­butadienes. Intrigued by a communication briefly mentioning the formation of the nitrile-functionalized compounds [Ph(C≡N)C=N—C(H)=C*X*_2_] (*X* = Cl or Br) by treatment of the α-amino­nitrile H_2_NCHPhC≡N with chloral or bromal (Sato & Adachi, 1978 ▸), we reinvestigated this reaction to explore the scope for the synthesis of other derivatives. For example, we succeeded in preparing the title compound [C_6_H_3_(OMe)_2_(C≡N)C=N—C(H)=CBr_2_], (1), bearing two electron-donating meth­oxy groups at the *meta*- and *para*-positions of the aryl ring (see Fig. 1 ▸).
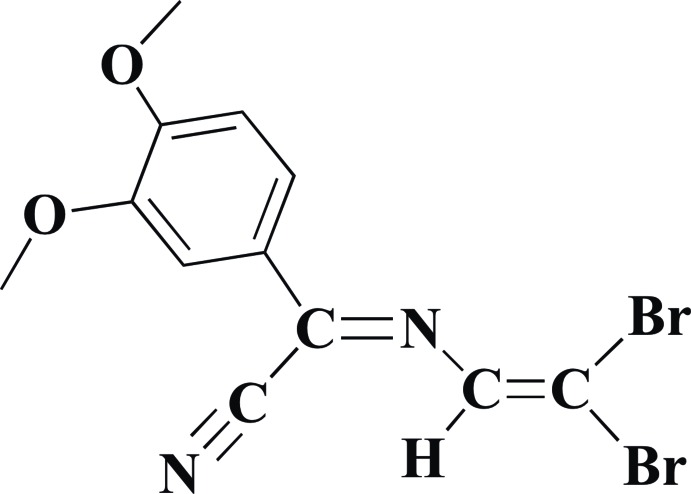


## Structural commentary   

**Figure 2:**
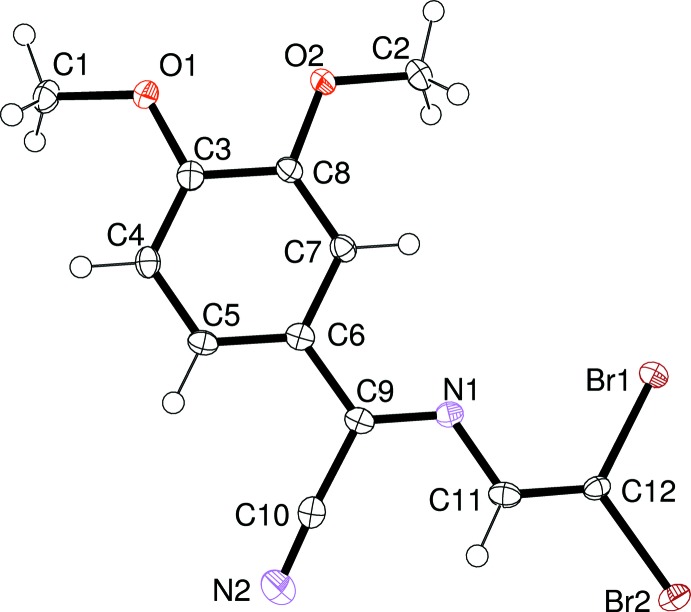
An displacement ellipsoid plot of (1) at the 50% probability level.

Compound (1) crystallizes from aceto­nitrile in the triclinic crystal system, space group *P* 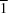. The *transoid* conformation of the aza­butadiene chain found in [Ph_2_C=N—C(H)=CCl_2_] (Jacquot *et al.*, 1999 ▸) is also observed in the crystal structure of (1) (Fig. 2 ▸). The aza­diene chain (C9/N1/C11/C12) is essentially planar (r.m.s. deviation = 0.014 Å). The torsion angle C12—C11—N1—C9 is 177.9 (3)°. The aryl ring, as well as the CN substituent, form part of the π-conjugated array. The length of the vinylic C11=C12 bond matches well with that of [Ph_2_C=N—C(H)=CCl_2_] [1.332 (4) *versus* 1.319 (3) Å]. We are not aware of any other structurally characterized aza­butadienes bearing a Br_2_C=C moiety. For other organic compounds containing this di­bromo­vinyl unit, such as 2,2-di­bromo­vinyl­thio­phene and 2-(2,2-di­bromo­vin­yl)-1-methyl-1*H*-imidazole-4,5-dicarbo­nitrile, C=C distances of 1.335 (7) and 1.317 (3) Å have been reported (Clément *et al.*, 2011 ▸; Lokaj *et al.*, 2011 ▸). The C9=N1 bond length of the imine group is also comparable with that of [Ph_2_C=N—C(H)=CCl_2_] [1.288 (3) *versus* 1.293 (2) Å].

## Supra­molecular features   

**Figure 3:**
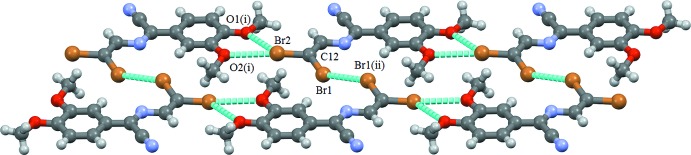
Part of the crystal structure of (1), showing the formation of double-wide ribbons through halogen C—Br⋯O and C—Br⋯Br—C bonding. [Symmetry codes: (i) *x* − 1, *y*, *z* − 1; (ii) −*x* + 1, −*y* + 2, −*z* + 1.]

**Figure 4:**
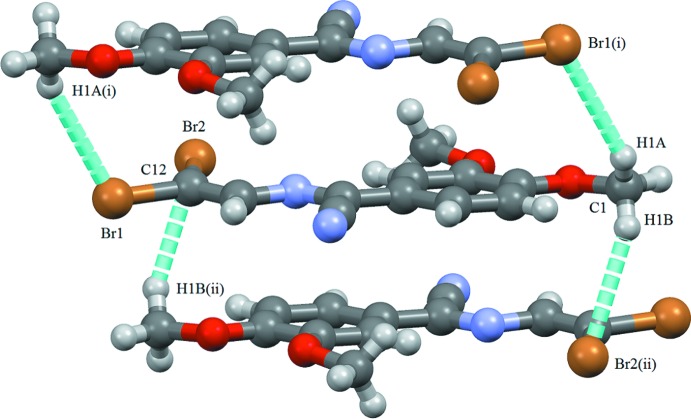
Part of the crystal structure of (1), showing the C—H⋯Br inter­actions. [Symmetry codes: (i) −*x* + 1, −*y* + 1, −*z* + 1; (ii) −*x*, −*y* + 1, −*z* + 1.]

**Figure 5:**
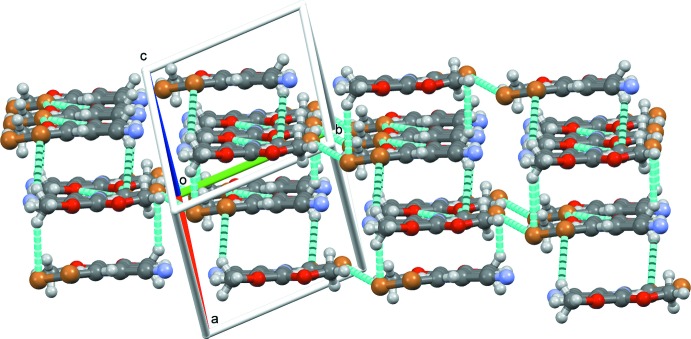
Part of the three-dimensional packing in (1) projected down the [101] direction and showing the halogen and weak C–H⋯Br inter­actions detailed in Figs. 3 ▸ and 4 ▸.

**Figure 6:**
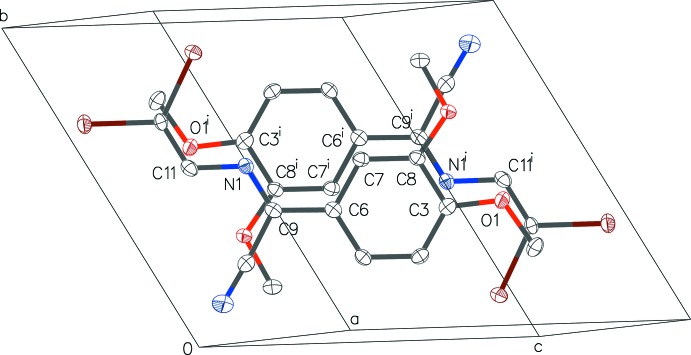
Part of the crystal structure of (1), showing the potential π–π inter­actions in two head-to-tail mol­ecules overlapping around the symmetry centre at (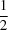, 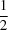, 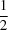) (see also Fig. 4 ▸). H atoms have been omitted for clarity. [Symmetry code: (i) −*x* + 1, −*y* + 1, −*z* + 1.]

**Figure 7:**
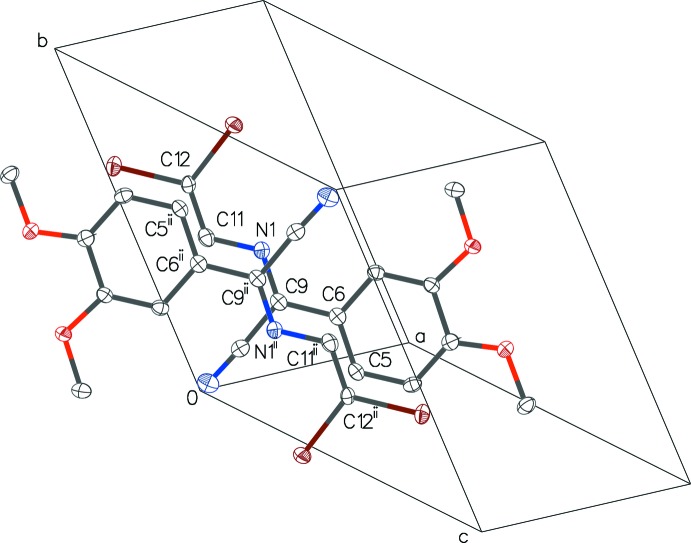
Part of the crystal structure of (1), showing the potential π–π inter­actions in two head-to-tail mol­ecules overlapping around the symmetry centre at (0, 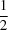, 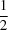) (see also Fig. 4 ▸). H atoms have been omitted for clarity. [Symmetry code: (ii) −*x*, −*y* + 1, −*z* + 1.]

Each planar mol­ecule of (1) is connected through halogen (Cavallo *et al.*, 2016 ▸) bifurcated bonds C12—Br2⋯(O1,O2) to two neighbouring mol­ecules to form a one-dimensional ribbon. The ribbon is further connected through another kind of side halogen bond (C12—Br1⋯Br1—C12) to other neighbouring mol­ecules with the formation of roughly planar one-dimensional double-wide straight chains (Fig. 3 ▸ and Table 1 ▸). These chains then stack in a slipped manner through very weak C—H⋯Br inter­actions (Fig. 4 ▸ and Table 2 ▸) to generate a three-dimensional supra­molecular network (Fig. 5 ▸). When projecting the structure down the direction perpendicular to the planes of the planar mol­ecules of (1) (*e.g.* down from the top in Fig. 4 ▸), one sees an inter­esting overlap in a head-to-tail arrangement of zigzagging unsaturated chains that leads to the formation of π–π stacking inter­actions around the symmetry centres located at (0, 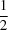, 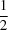) and (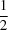, 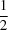, 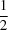). They consist of overlaps between the aza­diene C=C and C=N double bonds and parts of the aryl rings. For clarity, these overlaps are shown separately in Figs. 6 ▸ and 7 ▸. The mean inter­atomic separation between the chains built around (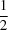, 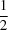, 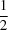) (Fig. 6 ▸ and Table 3 ▸) is 3.523 (5) Å, while a slightly shorter separation of 3.464 (5) Å is observed for the second couple built around (0, 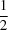, 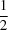) (Fig. 7 ▸ and Table 3 ▸).

## Database survey   

There are several other examples of structurally characterized 2-aza­butadienes bearing cyano (nitrile) substituents attached at the aza­butadienic array. These include 3-cyano-4-(*n*-meth­oxy­phen­yl)-1,1-diphenyl-2-aza-1,3-butadienes (*n* = 2, 3 or 4), 3-cyano-4-(4-cyano­phen­yl)-1,1-diphenyl-2-aza-1,3-butadiene, 3-cyano-4-(2,4-di­meth­oxy­phen­yl)-1,1-diphenyl-2-aza-1,3-butadiene, 3-cyano-4-(2,4-di­chloro­phen­yl)-1,1-diphenyl-2-aza-1,3-butadiene and 3-cyano-4-(*n*-fluoro­phen­yl)-1,1-diphenyl-2-aza-1,3-butadienes (*n* = 2 or 4) (Angelova *et al.*, 1993*a* ▸,*b* ▸; Macícek *et al.*, 1993*a* ▸,*b* ▸; Dryanska *et al.*, 1995 ▸). Furthermore, the structure of (*E*)-4,4-di­cyano-3-methyl­thio-1-phenyl-1-(1-pyr­rolidin­yl)-2-aza­buta-1,3-diene has been reported (Lorente *et al.*, 1996 ▸). Note that in all these structures there is a significant deviation from linearity of the C=N—C=C chain. This feature is due to the presence of a substituent at the 3-C position of the 2-aza­buta-1,3-diene chain. We also observed and discussed this feature in the structures of [Ar_2_C=N—C(S^t^Bu)=C(H)S^t^Bu] (Kinghat *et al.*, 2016 ▸).

## Synthesis and crystallization   

The required α-amino­nitrile used a starting material was obtained according a literature protocol (Mai & Patil, 1984 ▸). An equimolar mixture of *N*-(di­bromo­ethylen­yl)-1-imino-1-vertraceto­nitrile (10 mmol) and tri­bromo­acetaldehyde in 10 ml of aceto­nitrile was stirred under reflux for 2 h. The solution was then filtered and all volatiles removed under reduced pressure. The crude residue was recrystallized from aceto­nitrile affording clear-light orange crystals (yield 79%; m.p. 440 K; ^1^H RMN (CDCl_3_, 300 MHz): δ 3.95 (*s*, 3H, OCH_3_), 3.96 (*s*, 3H, OCH_3_), 6.93 (*d*, 1H, *J* = 9 Hz, 1 Ar-H), 7.65 (*s*, 2H, 2 Ar-H), 8.04 (*s*, 1H, =CH); ^13^C{^1^H} NMR (CDCl_3_, 75 MHz): δ 55.9 (O*C*H_3_), 56.2 (O*C*H_3_), 103.2 (=*C*Br_2_), 110.6 (*C*≡N), 124.3–153.9 (*C*_Ar_), 137.8 (*C*=N), 142.2 (*C*H); λ_max_ = 245 nm (e = 3300 M^−1^ cm^−1^), λ_max_ = 353 nm (e = 7580 M^−1^ cm^−1^); IR (ATR) cm^−1^: 2219 (C≡N), 1597 (C=N), 1569 (C=C).

## Refinement details   

Crystal data, data collection and structure refinement details are summarized in Table 4 ▸. All H atoms were placed in calculated positions and treated in a riding model. C—H distances were set at 0.95 (aromatic) and 0.98 Å (meth­yl), with *U*_iso_(H) = *xU*_eq_(C), where *x* = 1.5 for idealized methyl H atoms refined as rotating groups and 1.2 for all other H atoms.

## Supplementary Material

Crystal structure: contains datablock(s) I. DOI: 10.1107/S2056989016011075/pk2582sup1.cif

Structure factors: contains datablock(s) I. DOI: 10.1107/S2056989016011075/pk2582Isup2.hkl

Supporting information file. DOI: 10.1107/S2056989016011075/pk2582Isup3.cml

CCDC reference: 1491488

Additional supporting information: crystallographic information; 3D view; checkCIF report

## Figures and Tables

**Table 1:** Halogen-bonding parameters (Å, °) for (1)

*D*	Br	*A*	*D*—Br	Br⋯*A*	D—Br⋯A
C12	Br2	O1^i^	1.878 (3)	3.185 (2)	124.26 (9)
C12	Br2	O2^i^	1.878 (3)	3.153 (2)	167.6 (1)
C12	Br1	Br1^ii^	1.872 (3)	3.4340 (5)	144.8 (1)

Symmetry codes: (i) *x* − 1, *y*, *z* − 1; (ii) −*x* + 1, −*y* + 2, −*z* + 1.

**Table 2:** Hydrogen-bond geometry (Å, °)

*D*—H⋯*A*	*D*—H	H⋯*A*	*D*⋯*A*	*D*—H⋯*A*
C1—H1*A*⋯Br1^i^	0.98	3.01	3.867 (4)	146
C1—H1*B*⋯Br2^ii^	0.98	3.04	3.869 (4)	143

Symmetry codes: (i) 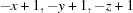; (ii) 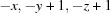.

**Table 3:** π–π inter­actions (Å) in (1)

Atom *A*	Atom *B*	*A*⋯*B*	Atom *C*	Atom *D*	*C*⋯D
C5	C12^ii^	3.445 (5)	C11	O1^i^	3.455 (4)
C6	C11^ii^	3.497 (5)	N1	C3^i^	3.556 (4)
C9	N1^ii^	3.451 (4)	C9	C8^i^	3.523 (5)
			C6	C7^i^	3.559 (5)

Symmetry codes: (i) 1 − *x*, 1 − *y*, 1 − *z*; (ii) −*x*, 1 − *y*, 1 − *z*.

**Table 4:** Experimental details

Crystal data	
Chemical formula	C_12_H_10_Br_2_N_2_O_2_
*M*_r_	374.04
Crystal system, space group	Triclinic, *P* 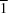
Temperature (K)	100
*a*, *b*, *c* (Å)	7.6878 (4), 9.2782 (5), 10.8111 (6)
α, β, γ (°)	106.162 (2), 100.887 (2), 110.009 (2)
*V* (Å^3^)	660.57 (6)
*Z*	2
Radiation type	Mo *K*α
μ (mm^−1^)	6.13
Crystal size (mm)	0.25 × 0.2 × 0.1

Data collection	
Diffractometer	Bruker D8 VENTURE
Absorption correction	Multi-scan (*SADABS*; Bruker, 2014 ▸)
*T*_min_, *T*_max_	0.537, 0.746
No. of measured, independent and observed [*I* > 2σ(*I*)] reflections	23955, 3045, 2442
*R*_int_	0.067
(sin θ/λ)_max_ (Å^−1^)	0.652

Refinement	
*R*[*F* ^2^ > 2σ(*F* ^2^)], *wR*(*F* ^2^), *S*	0.027, 0.067, 1.03
No. of reflections	3045
No. of parameters	165
H-atom treatment	H-atom parameters constrained
Δρ_max_, Δρ_min_ (e Å^−3^)	0.80, −0.42

Computer programs: *APEX2* and *SAINT* (Bruker, 2013 ▸), *SHELXT* (Sheldrick, 2015*a* ▸), *SHELXL2014* (Sheldrick, 2015*b* ▸) and *OLEX2* (Dolomanov *et al.*, 2009 ▸).

## supplementary crystallographic information

### Crystal data

**Table:**

C_12_H_10_Br_2_N_2_O_2_	*Z* = 2
*M**_r_* = 374.04	*F*(000) = 364
Triclinic, *P*1	*D*_x_ = 1.881 Mg m^−^^3^
*a* = 7.6878 (4) Å	Mo *K*α radiation, λ = 0.71073 Å
*b* = 9.2782 (5) Å	Cell parameters from 8732 reflections
*c* = 10.8111 (6) Å	θ = 3.0–27.5°
α = 106.162 (2)°	µ = 6.13 mm^−^^1^
β = 100.887 (2)°	*T* = 100 K
γ = 110.009 (2)°	Plqte, clear light orange
*V* = 660.57 (6) Å^3^	0.25 × 0.2 × 0.1 mm

### Data collection

**Table:**

Bruker D8 VENTURE diffractometer	3045 independent reflections
Radiation source: X-ray tube, Siemens KFF Mo 2K-90C	2442 reflections with *I* > 2σ(*I*)
TRIUMPH curved crystal monochromator	*R*_int_ = 0.067
φ and ω scans'	θ_max_ = 27.6°, θ_min_ = 3.0°
Absorption correction: multi-scan (SADABS; Bruker, 2014)	*h* = −9→10
*T*_min_ = 0.537, *T*_max_ = 0.746	*k* = −12→12
23955 measured reflections	*l* = −14→14

### Refinement

**Table:**

Refinement on *F*^2^	Primary atom site location: structure-invariant direct methods
Least-squares matrix: full	Hydrogen site location: inferred from neighbouring sites
*R*[*F*^2^ > 2σ(*F*^2^)] = 0.027	H-atom parameters constrained
*wR*(*F*^2^) = 0.067	*w* = 1/[σ^2^(*F*_o_^2^) + (0.0307*P*)^2^ + 0.5386*P*] where *P* = (*F*_o_^2^ + 2*F*_c_^2^)/3
*S* = 1.03	(Δ/σ)_max_ = 0.001
3045 reflections	Δρ_max_ = 0.80 e Å^−^^3^
165 parameters	Δρ_min_ = −0.42 e Å^−^^3^
0 restraints	

### Special details

**Table:**

Experimental. Absorption correction: SADABS-2014/4 (Bruker,2014) was used for absorption correction. wR2(int) was 0.0938 before and 0.0647 after correction. The Ratio of minimum to maximum transmission is 0.7197. The λ/2 correction factor is 0.00150.
Geometry. All esds (except the esd in the dihedral angle between two l.s. planes) are estimated using the full covariance matrix. The cell esds are taken into account individually in the estimation of esds in distances, angles and torsion angles; correlations between esds in cell parameters are only used when they are defined by crystal symmetry. An approximate (isotropic) treatment of cell esds is used for estimating esds involving l.s. planes.

### Fractional atomic coordinates and isotropic or equivalent isotropic displacement parameters (Å^2^)

**Table:**

	*x*	*y*	*z*	*U*_iso_*/*U*_eq_	
C1	0.5229 (4)	0.2556 (4)	0.9031 (3)	0.0198 (6)	
H1A	0.5402	0.1716	0.8346	0.030*	
H1B	0.3876	0.2129	0.9037	0.030*	
H1C	0.6109	0.2824	0.9927	0.030*	
C2	0.6815 (5)	0.8341 (4)	0.8299 (3)	0.0246 (7)	
H2A	0.7114	0.8389	0.7465	0.037*	
H2B	0.7909	0.9196	0.9088	0.037*	
H2C	0.5636	0.8527	0.8307	0.037*	
C3	0.4518 (4)	0.3908 (3)	0.7558 (3)	0.0163 (6)	
C4	0.3008 (4)	0.2473 (3)	0.6590 (3)	0.0160 (6)	
H4	0.2729	0.1450	0.6709	0.019*	
C5	0.1904 (4)	0.2535 (3)	0.5444 (3)	0.0156 (6)	
H5	0.0857	0.1552	0.4792	0.019*	
C6	0.2308 (4)	0.4007 (3)	0.5242 (3)	0.0149 (5)	
C7	0.3852 (4)	0.5476 (3)	0.6221 (3)	0.0154 (6)	
H7	0.4130	0.6496	0.6096	0.019*	
C8	0.4946 (4)	0.5419 (3)	0.7352 (3)	0.0149 (5)	
C9	0.1186 (4)	0.4092 (3)	0.4029 (3)	0.0148 (5)	
C10	−0.0396 (4)	0.2543 (4)	0.3038 (3)	0.0210 (6)	
C11	0.0555 (4)	0.5495 (3)	0.2655 (3)	0.0175 (6)	
H11	−0.0400	0.4495	0.1958	0.021*	
C12	0.0911 (4)	0.6927 (3)	0.2490 (3)	0.0139 (5)	
N1	0.1576 (3)	0.5463 (3)	0.3839 (2)	0.0145 (5)	
N2	−0.1661 (4)	0.1374 (3)	0.2229 (3)	0.0356 (7)	
O1	0.5666 (3)	0.4013 (2)	0.8715 (2)	0.0195 (4)	
O2	0.6500 (3)	0.6752 (2)	0.8351 (2)	0.0195 (4)	
Br1	0.27146 (4)	0.89631 (3)	0.38235 (3)	0.01976 (9)	
Br2	−0.04365 (4)	0.69810 (3)	0.08743 (3)	0.01900 (9)	

### Atomic displacement parameters (Å^2^)

**Table:**

	*U*^11^	*U*^22^	*U*^33^	*U*^12^	*U*^13^	*U*^23^
C1	0.0209 (14)	0.0221 (14)	0.0233 (16)	0.0103 (12)	0.0097 (12)	0.0148 (13)
C2	0.0258 (16)	0.0142 (14)	0.0254 (17)	0.0023 (12)	0.0012 (13)	0.0074 (13)
C3	0.0145 (13)	0.0177 (14)	0.0202 (15)	0.0082 (11)	0.0082 (11)	0.0086 (12)
C4	0.0180 (14)	0.0152 (13)	0.0194 (15)	0.0087 (11)	0.0095 (12)	0.0083 (11)
C5	0.0121 (13)	0.0167 (13)	0.0147 (14)	0.0043 (11)	0.0038 (11)	0.0035 (11)
C6	0.0142 (12)	0.0161 (13)	0.0146 (14)	0.0071 (10)	0.0062 (11)	0.0038 (11)
C7	0.0168 (13)	0.0137 (13)	0.0144 (14)	0.0050 (11)	0.0058 (11)	0.0044 (11)
C8	0.0132 (13)	0.0142 (13)	0.0152 (14)	0.0039 (10)	0.0046 (11)	0.0048 (11)
C9	0.0133 (13)	0.0151 (13)	0.0146 (14)	0.0061 (10)	0.0055 (11)	0.0025 (11)
C10	0.0226 (15)	0.0186 (14)	0.0217 (16)	0.0076 (13)	0.0050 (13)	0.0099 (13)
C11	0.0171 (14)	0.0170 (14)	0.0141 (14)	0.0069 (11)	0.0033 (11)	0.0012 (11)
C12	0.0133 (12)	0.0179 (13)	0.0104 (13)	0.0064 (11)	0.0042 (11)	0.0053 (11)
N1	0.0157 (11)	0.0160 (11)	0.0117 (12)	0.0072 (9)	0.0049 (9)	0.0038 (9)
N2	0.0337 (16)	0.0205 (14)	0.0371 (18)	0.0036 (12)	−0.0042 (14)	0.0087 (13)
O1	0.0191 (10)	0.0185 (10)	0.0202 (11)	0.0059 (8)	0.0025 (8)	0.0110 (9)
O2	0.0203 (10)	0.0130 (9)	0.0174 (11)	0.0017 (8)	−0.0006 (8)	0.0053 (8)
Br1	0.02102 (15)	0.01428 (14)	0.01661 (16)	0.00322 (11)	0.00125 (12)	0.00357 (11)
Br2	0.02026 (15)	0.02270 (16)	0.01424 (16)	0.01035 (12)	0.00265 (12)	0.00730 (12)

### Geometric parameters (Å, º)

**Table:**

C1—H1A	0.9800	C5—C6	1.383 (4)
C1—H1B	0.9800	C6—C7	1.415 (4)
C1—H1C	0.9800	C6—C9	1.464 (4)
C1—O1	1.431 (3)	C7—H7	0.9500
C2—H2A	0.9800	C7—C8	1.372 (4)
C2—H2B	0.9800	C8—O2	1.372 (3)
C2—H2C	0.9800	C9—C10	1.465 (4)
C2—O2	1.430 (3)	C9—N1	1.288 (3)
C3—C4	1.387 (4)	C10—N2	1.147 (4)
C3—C8	1.418 (4)	C11—H11	0.9500
C3—O1	1.345 (3)	C11—C12	1.332 (4)
C4—H4	0.9500	C11—N1	1.384 (4)
C4—C5	1.391 (4)	C12—Br1	1.872 (3)
C5—H5	0.9500	C12—Br2	1.878 (3)
			
H1A—C1—H1B	109.5	C5—C6—C9	121.7 (2)
H1A—C1—H1C	109.5	C7—C6—C9	118.7 (2)
H1B—C1—H1C	109.5	C6—C7—H7	120.2
O1—C1—H1A	109.5	C8—C7—C6	119.6 (3)
O1—C1—H1B	109.5	C8—C7—H7	120.2
O1—C1—H1C	109.5	C7—C8—C3	120.5 (3)
H2A—C2—H2B	109.5	O2—C8—C3	114.7 (2)
H2A—C2—H2C	109.5	O2—C8—C7	124.8 (2)
H2B—C2—H2C	109.5	C6—C9—C10	117.0 (2)
O2—C2—H2A	109.5	N1—C9—C6	121.6 (2)
O2—C2—H2B	109.5	N1—C9—C10	121.5 (3)
O2—C2—H2C	109.5	N2—C10—C9	176.7 (3)
C4—C3—C8	119.4 (3)	C12—C11—H11	120.0
O1—C3—C4	125.4 (3)	C12—C11—N1	120.0 (3)
O1—C3—C8	115.1 (2)	N1—C11—H11	120.0
C3—C4—H4	120.1	C11—C12—Br1	123.0 (2)
C3—C4—C5	119.8 (3)	C11—C12—Br2	120.4 (2)
C5—C4—H4	120.1	Br1—C12—Br2	116.65 (14)
C4—C5—H5	119.5	C9—N1—C11	120.4 (2)
C6—C5—C4	120.9 (3)	C3—O1—C1	118.0 (2)
C6—C5—H5	119.5	C8—O2—C2	116.8 (2)
C5—C6—C7	119.6 (3)		
			
C3—C4—C5—C6	1.1 (4)	C7—C6—C9—C10	179.8 (2)
C3—C8—O2—C2	−171.0 (2)	C7—C6—C9—N1	0.0 (4)
C4—C3—C8—C7	1.5 (4)	C7—C8—O2—C2	9.3 (4)
C4—C3—C8—O2	−178.2 (2)	C8—C3—C4—C5	−1.4 (4)
C4—C3—O1—C1	−4.4 (4)	C8—C3—O1—C1	175.8 (2)
C4—C5—C6—C7	−0.7 (4)	C9—C6—C7—C8	−178.7 (2)
C4—C5—C6—C9	178.7 (3)	C10—C9—N1—C11	−2.9 (4)
C5—C6—C7—C8	0.8 (4)	C12—C11—N1—C9	177.2 (3)
C5—C6—C9—C10	0.3 (4)	N1—C11—C12—Br1	−1.7 (4)
C5—C6—C9—N1	−179.5 (3)	N1—C11—C12—Br2	179.0 (2)
C6—C7—C8—C3	−1.2 (4)	O1—C3—C4—C5	178.8 (3)
C6—C7—C8—O2	178.5 (2)	O1—C3—C8—C7	−178.7 (3)
C6—C9—N1—C11	176.9 (2)	O1—C3—C8—O2	1.5 (4)

### Hydrogen-bond geometry (Å, º)

**Table:**

*D*—H···*A*	*D*—H	H···*A*	*D*···*A*	*D*—H···*A*
C1—H1*A*···Br1^i^	0.98	3.01	3.867 (4)	146
C1—H1*B*···Br2^ii^	0.98	3.04	3.869 (4)	143

Symmetry codes: (i) −*x*+1, −*y*+1, −*z*+1; (ii) −*x*, −*y*+1, −*z*+1.
